# Marked exposure to the endocrine-disrupting chemicals phthalates and bisphenol A in the ICU

**DOI:** 10.1186/cc14444

**Published:** 2015-03-16

**Authors:** J Huygh, P Jorens

**Affiliations:** 1 Antwerp University Hospital, Edegem, Belgium

## Introduction

Care for ICU patients has benefited from medical devices. Bisphenol A (BPA) and phthalates can leach from the plastic matrix. We hypothesized that ICU patients are exposed to BPA and phthalates through medical devices.

## Methods

Serum (*n *= 118) and urinary (*n *= 102) samples of adult (*n *= 35) ICU patients were analyzed for total BPA and di(2-ethylhexyl)phthalate (DEHP) and other phthalate metabolites (PMs). We also enrolled patients preoperatively before scheduled thoracic surgery and repeat samples were taken on days 1 to 4 during the ICU stay. Control data came from 44 healthy controls or from referenced literature.

## Results

Our Results show that adult ICU patients are continuously exposed to phthalates (that is, DEHP) as well as to BPA, albeit to a lesser extent, resulting in detectable serum and urinary levels in almost every patient and at every studied time point. Moreover, these levels were significantly higher than in controls or compared with referenced literature. The chronology of exposure was demonstrated: the preoperative urine and serum levels of the DEHP metabolites were often below the detection limit. Medical devices are the source of these chemicals: patients on hemofiltration, extracorporeal membrane oxygenation or both showed serum levels 100-fold or 1,000-fold higher than the general population or workers in plastic industry. The serum and some of the urinary levels of the DEHP metabolites are the highest ever reported in humans; some at biologically highly relevant concentrations of even ≥10 to 50 μM.

## Conclusion

Adult ICU patients are exposed to plastic softeners, in particular PMs. Despite the continuously tightening regulations, BPA and DEHP are still present in medical devices. Because patient safety is a concern in the ICU, further research into the (possibly toxic and clinical) effects of chemicals released from medical devices should be undertaken.

